# Key components of external facilitation in an acute stroke quality improvement collaborative in the Veterans Health Administration

**DOI:** 10.1186/s13012-015-0252-y

**Published:** 2015-05-14

**Authors:** Balmatee Bidassie, Linda S Williams, Heather Woodward-Hagg, Marianne S Matthias, Teresa M Damush

**Affiliations:** VA Center for Applied Systems Engineering, VISN11 - Veterans Engineering Resource Center, Roudebush VAMC, Indianapolis, IN USA; HSR&D VA Stroke QUERI Center and VA Center for Health Information and Communication (CHIC), Roudebush VAMC, Regenstrief Inc, Indiana University School of Medicine, Indianapolis, IN USA; Departments of Communication, General Internal Medicine and Geriatrics, and Neurology, Indiana University School of Medicine, Indianapolis, IN USA

**Keywords:** External facilitation, Implementation science, Stroke quality improvement, Collaborative

## Abstract

**Background:**

Facilitation is a key component for successful implementation in several implementation frameworks; however, there is a paucity of research specifying this component. As part of a stroke quality improvement intervention in the Veterans Health Administration (VHA), facilitation plus data feedback was compared to data feedback alone in 11 VA medical facilities. The objective of this study was to elucidate upon the facilitation components of the stroke quality improvement.

**Methods:**

We conducted a secondary evaluation of external facilitation using semi-structured interviews. Five facilitators and two program directors were interviewed. Qualitative analysis was performed on transcribed interviews to gain an understanding of the role and activities of external facilitators during the on-site and telephone facilitation. Quantitative frequencies were calculated from the self-reported time spent in facilitation tasks by facilitators.

**Results:**

The external facilitators saw their role as empowering the clinical teams to take ownership of the process changes at the clinical sites to improve their performance quality. To fulfill this role, they reported engaging in a number of core tasks during telephone and on-site visits including: assessing the context in which the teams were currently operating, guiding the clinical teams through their planned changes and use of process improvement tools, identifying resources and making referrals, holding teams accountable for plan implementation with on-site visits, and providing support and encouragement to the teams. Time spent in facilitation activities changed across time from guiding change (early) to supporting efforts made by the clinical teams (later). Facilitation activity transitioned to more monitoring, problem solving, and intentional work to hand over the clinical improvement process to the site teams with the coach’s role being increasingly that of a more distant consultant. Overall, this study demonstrated that external facilitation is not an event but rather a process where relationships and responsibilities evolve over time.

**Conclusions:**

This study shows that external facilitation involves core elements related to communication, relationship building, methods training, monitoring performance over time, and facilitating team-based problem solving. Importantly, this work demonstrates the fluid nature of external facilitation over time, as teams learn, grow, change, and experience changing contexts.

**Electronic supplementary material:**

The online version of this article (doi:10.1186/s13012-015-0252-y) contains supplementary material, which is available to authorized users.

## Background

Despite the fact that facilitation has yet to be operationally defined or well-evaluated, particularly within multi-site developments across a health system, it has been described as a process of interactive problem solving and support that enables the implementation of evidence into practice and is viewed as a key construct of successful implementation in a complex improvement environment like healthcare [1,2]. More specifically, successful implementation is posited to occur when implementation processes are appropriately facilitated by internal (e.g., inside the local site) and/or external (e.g., outside of the local site) facilitators [1]. Facilitation is a major component of the Promoting Action on Research Implementation in Health Services (PARiHS) framework [1,3,4]. Developed in the 1990s as a conceptual framework, PARiHS proposes that successful implementation of evidence into practice is a function of three broad interactive elements (evidence, context, and facilitation) [5,6]. Successful implementation [7] is most likely to occur when 1) scientific evidence is viewed as sound and fitting with professional and patient beliefs; 2) the healthcare context is receptive to implementation in terms of supportive leadership, culture, and evaluative systems [1,8]; and 3) there are appropriate mechanisms in place to facilitate implementation. Facilitation (external/internal) is considered a complex and multifaceted concept [1]; however, there exists little research which specifies the necessary components of facilitation for successful implementation.

Recently, an application of facilitation was illustrated in a partnered facilitation strategy using both external and internal facilitators to assist clinics in the Veterans Health Administration (VHA) to implement a primary care-mental health integration service [9,10]. Three external facilitators spent 3,955 hours helping 19 clinics and internal facilitators implement the targeted practice that resulted in greater reach and adoption of a mandated initiative [10]. Most frequent activities of the facilitators were preparation and planning, stakeholder engagement, and education. While this application provides specificity on the facilitation role, there is a lack of a consistent operational definition of facilitation in the literature and a lack of specificity on the role to be deemed as appropriate and sufficient to enable successful implementation by the targeted users of a healthcare practice.

Recently, facilitation was incorporated into a bundled stroke quality improvement project across 11 Veterans Affair (VA) Medical Centers. To improve the quality of acute stroke services [11] in the Veterans Health Administration (VHA), the VA stroke Quality Enhancement Research Initiative (QUERI) [12] partnered with VA Center for Applied Systems Engineering (VA-CASE) [13], an interdisciplinary Veterans Engineering Resource Center (VERC) to provide industrial/systems engineering facilitation and tools to evaluate a stroke learning collaborative [14-16] intervention. Given the complexity of the acute stroke clinical practice changes and the training literature [16], the collaborative program training [17] alone is insufficient to produce and sustain practice changes [18]. Therefore, telephone facilitation is an important and efficient approach to provide facilitation service [19]. The Stroke-QUERI facilitators (implementation research facilitators) and the Industrial Engineer (IE) facilitators from VA-CASE provided external facilitation to the clinical stroke teams after the collaborative learning meeting when the teams returned to their medical facilities.

IE facilitators are used in other VA clinical collaborative learning initiatives [20-22], and Stroke-QUERI facilitators have experience providing external facilitation to the clinicians in the field [2], but relatively little is known about what the external facilitators actually do as intended in the PARiHS framework during implementation of QI with the clinical teams [23].

The specific aim of this project was to elucidate upon the facilitation techniques that were used within the front line stroke clinicians and teams to promote stroke quality improvement. We examined facilitation in-depth including activities, processes, and challenges of external facilitation. Specifically, we identified the core components of facilitation by telephone and on-site and evaluated changes in these activities over time in an in-hospital stroke quality improvement study.

## Methods

### Design

This qualitative study was a secondary analysis of a clustered, randomized controlled trial of a stroke quality improvement intervention [24]. The Intervention for Stroke Improvement using Redesign Engineering (INSPIRE) study compared a stroke collaborative learning in operational systems engineering methods followed by 6 months of external facilitation along with audit and feedback on acute stroke care quality performance (five facilities) compared to acute stroke care quality performance feedback alone (six facilities) to improve two stroke processes of care (venous thromboembolism prevention and dysphagia screening) (Williams LS, Daggett V, Slaven J, Zhangsheng Y, Sager D, Myers J, Plue L, Woodward-Hagg H, Damush TM: A cluster-randomized trial to improve two inpatient stroke quality indicators, submitted). Clinical stroke teams in the intervention received training in a face-to-face collaborative. Implementation researchers from the Stroke-QUERI and IEs from VA-CASE designed and participated in the training sessions. Prior to the collaborative, a Stroke-QUERI coach and an IE coach were paired and assigned to provide external facilitation [25] to the clinical stroke teams during the collaborative and for an additional 6 months of facilitation consisting of one to two site visits and at least bi-weekly conference calls with the local teams. The two types of facilitators were paired into teams so that their combined skills (IE process improvement skills and tools and Stroke-QUERI clinical skills and familiarity with stroke quality processes) could provide optimal resources to obtain process changes that could be sustained at the VA facilities.

**Figure 1:**
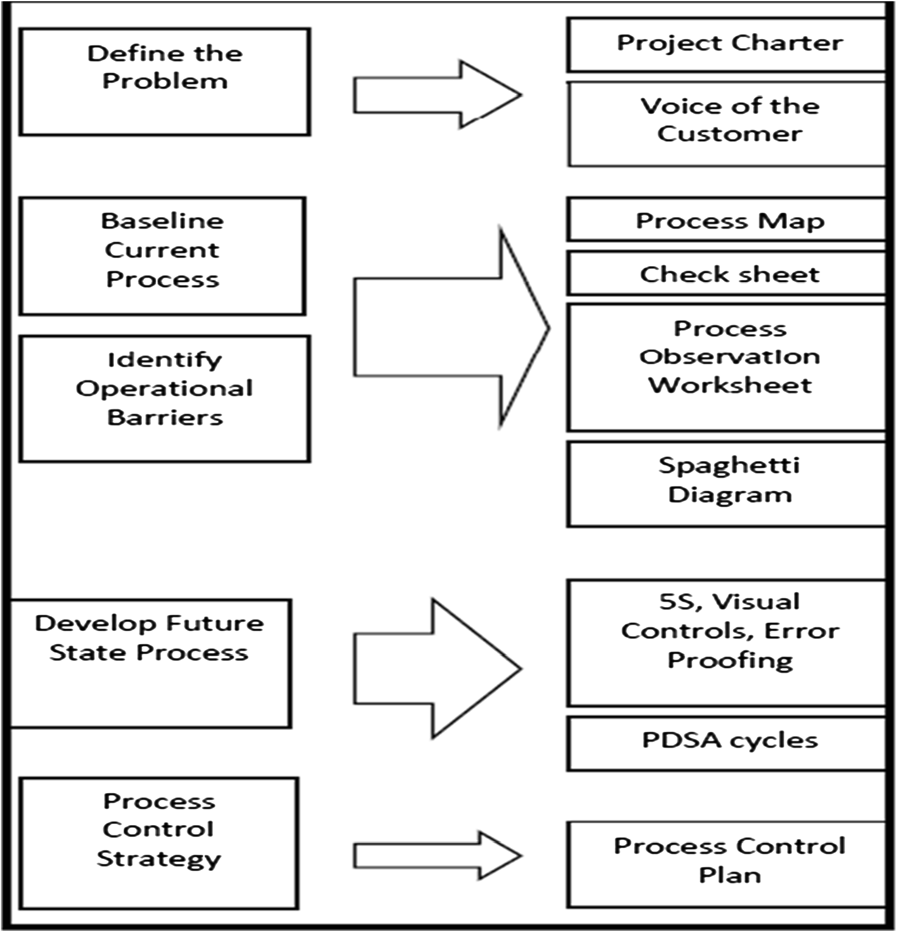
Roadmap of process improvement tools provided to teams to facilitate quality improvement [21].

The facilitation strategy promoted by VA-CASE uses a combination of both developmental and process facilitation, working from within the group and focusing on the processes for change [21]. When working with clinical teams, the VA-CASE method emphasizes the principles and the tools provided to teams to facilitate quality improvement (see Figure 1). The Stroke-QUERI facilitators followed a case-management approach applying problem solving and goal-setting methods with front line clinicians.

During the learning collaborative, the Stroke-QUERI facilitators provided training on the stroke quality protocols and measurement and the IE facilitators provided process improvement support, applied systems redesign (SR) [26] operational systems engineering (OSE) principles, and training to the teams [2]. Table 1 lists process improvement tools that were utilized during the learning collaborative and subsequently in the facilitation processes.

**Table 1:** **Providing tool support to the stroke teams to facilitate quality improvement**

	**Self-reported % of time spent on encouraging application of process improvement tools (** ***n*** **= 5)**				
**Process improvement tools**	**Stroke-QUERI facilitators**			**Industrial engineer (IE) facilitators**	
	**(Implementation researchers)**			**(VERC VA-CASE)**	
	**022001**	**022003**	**022005**	**022002**	**022004**
• Project charter	25%	20%	30%	25%	20%
• Process map	15%	10%	50%	25%	80%
• Spaghetti diagram	15%	10%	10%	10%	15%
• Impact effort matrix	15%	10%	20%–40%	10%	0%
• PDSA (plan, do, study, act) cycles	80%	20%	100%	75%	80%

Note: process improvement tools were introduced to the stroke teams during the 3-day collaborative meeting and subsequently, during the facilitation sessions. Each facilitator was asked to estimate time from 0%–100% for each tool; therefore, their total time may be more or less than 100%.

The intervention sites received 6 months of external facilitation post collaborative and therefore provided an opportunity to illuminate the facilitation process. Specifically for this secondary analysis, all of the external facilitators were interviewed using semi-structured guides (see Additional file 1) at the end of all external facilitation activities to understand their unique and overlapping facilitation skills, knowledge, and contributions as well as identify the critical elements of external facilitation.

### Participants

The external facilitators included three Stroke-QUERI Facilitators (licensed social worker, registered nurse, social scientist) with specialized knowledge and skills in case management and implementation and two IE facilitators (IE, registered nurse) who were trained in Lean quality improvement methodology [21], an effective strategy for improving healthcare delivery processes. The paired facilitation teams (Stroke-QUERI and IE) were maintained throughout the project and they provided facilitation to the front line clinical stroke teams that existed at 11 VA medical centers across the United States. The facilitators logged notes after each facilitation call and on-site visit. During the collaboration, facilitators met several times with the steering committee which included an experienced coach facilitator and a stroke clinical quality improvement expert to review and discuss the progress and concerns of the facilitation roles, telephone calls, and on-site visits.

A qualitative interviewer with expertise in healthcare communications and no involvement with the INSPIRE intervention completed semi-structured interviews with all external facilitators. In addition, the leadership of both VA-CASE (an IE) and the Stroke-QUERI Centers (a neurologist) who also led the INSPIRE project were also interviewed.

### Measurement

The semi-structured questions (see Additional file 1) for the external facilitators were constructed to provide an in-depth evaluation of external facilitation for this active implementation intervention. The interview included domains related to the expectations and knowledge, tasks performed across the intervention phase, application of SR process, process improvement tools, joint facilitation experiences, and the enablers and barriers of external facilitation. Additional file 2 lists the interview guide for the leadership. All interviews were conducted face-to-face, were audiotaped and transcribed by an approved contractor. Each transcription was stripped of any identifiers and was assigned a unique identification number.

### Analysis plan

Each coder individually read the initial seven transcripts multiple times and extracted major themes (codes). All three coders (BB, LW, and TD) met as a team to review the individual codes and reach a consensus. The codebook was entered into NVIVO 10 software and all transcripts were coded using the codebook as well as additional emergent themes.

Following the development of the codebook, two coders worked independently (but simultaneously) to read each transcript attaching the relevant codes to selected sections of text. In essence, this process served to index the transcript by evaluating each line of text and attaching codes when the discussion content matches a category in the codebook. Any line in a transcript could be marked by an unlimited number of codes, if that section was relevant to more than one topic of interest. As a quality control measure, the two coders’ transcripts were merged and the results were compared. Duplicative codes collapsed into a single code while unique codes across the two reviewers were discussed and included in the merged file if deemed relevant. Once the coding was completed, reports of coding themes and frequencies were generated in NVIVO.

## Results

We present the identification of facilitation activities by on-site visits and by telephone followed by changes in activities over time working with the clinical teams. Next, we note differences by facilitator type and overall challenges to facilitation followed by major themes from the facilitators on their roles. Finally, we present the observations made by the program leaders.

### External facilitation identified activities

Table 1 shows that the time spent on the various facilitation tasks and tools utilized varied across the facilitators. Also, Tables 2 and 3 show this variation across the type of facilitation delivery (on-site visit vs telephone).

**Table 2:** **External facilitation tasks during on-site visits**

	**% of time reportedly spent on facilitation tasks (on-site visits) (** ***n***?**=?5)**				
**Facilitation tasks**	**Stroke-QUERI facilitators**			**Industrial engineer (IE) facilitators**	
	**(Implementation researchers)**			**(VERC VA-CASE)**	
	**022001**	**022003**	**022005**	**022002**	**022004**
a. Monitoring the status of the teams’ progress	~5%	~25%	40%–50%	50%	60%
b. Providing information to the teams	25%	~10%	30%	25%	15%–20%
c. Providing support (being a sound board, providing guidance)	10%	~75%	75%–80%	25%	50%
d. Identifying activities to be done	25%	25%	50%–60%	50%	30%–35%
e. Providing referrals (connecting people to people, connecting people to information	15%	5%	25%	25%	20%
f. Identifying resources	5%–10%	~5%	30%	25%	0%

Note: each facilitator was asked to estimate time from 0%–100% for each task; therefore, their total time may be more or less than 100%.

**Table 3:** **External facilitation during telephone sessions**

	**% of time reportedly spent on facilitation tasks (telephone sessions) (** ***n*** **= 5)**				
**Facilitation tasks**	**Stroke-QUERI facilitators**			**Industrial engineer (IE) facilitators**	
	**(Implementation researchers)**			**(VERC VA-CASE)**	
	**022001**	**022003**	**022005**	**022002**	**022004**
a. Monitoring the status of the teams’ progress	25%	50%	50%	75%	30%
b. Providing information to the teams	25%–30%	<10%	40% earlier and 15%–20% toward end	25%	50%
c. Providing support (being a sound board, providing guidance)	10%–15%	80%–90%	80%	25%	80%
d. Identifying activities to be done	25	~5%	45% earlier and 25% toward end	75%	
e. Providing referrals (connecting people to people, connecting people to information	10%	5%	40%–50% earlier and 10% toward end	25%	
f. Identifying resources	10%	5%	20% earlier and 10% toward end	25%	0%

Note: each facilitator was asked to estimate time from 0%–100% for each task; therefore, their total time may be more or less than 100%.

#### On-site visit

During on-site visits, the facilitators identified relationship building, team assessment, problem solving, and support as key tasks they used to engage the stroke clinical teams at the hospitals.

### Relationship building

The facilitators reported, as shown in Table 2, that establishing a good relationship with the teams was their key strategy; however, each approach was different. Some facilitators focused on having frequent contact with their teams and building a level of trust, then providing subsequent support, promoting the use of tools, role playing at the local facility, and then finally focused on implementation of changes. One facilitator began the facilitation experience by building a relationship with the clinical teams. “*My approach…I try to treat it the same way as a patient. I’m more in a relationship. I think you have to have a level of trust and start a relationship and kind of go from there…That’s probably my first strategy I use as a facilitator*”. Other facilitators started with the process and the tools to engage the clinical teams at the facilities. Their approach was to try to build project ownership by the team versus doing things for them. Most of the facilitators emphasized the importance of teamwork.

### Team assessment

During the on-site visits, the most important task for facilitators was the contextual assessment of the teams’ status at baseline prior to the initiation of stroke quality improvement. The facilitators talked with teams and looked at site quality performance, current processes, and structures and networks to figure out their status in terms of what existing programs and efforts around acute stroke care and quality improvement existed. The facilitators stated they preferred in-person visits to help them understand where the teams were and what they could do to help. As one explained, “(We communicated) *through more private conversations than group conversations, because one site did not want to share anything negative. But they were willing to share it with you one-on-one*”. On-site information enabled the facilitators to accurately frame the clinical team’s starting point and helped clarify existing interdisciplinary dynamics. The facilitators tended to use tools to figure out the progresses of their teams based upon the original PDSA (plan, do, study, act) cycles and project charter intentions. In addition, the facilitators determined the gaps in teams’ performances and helped them to plan on how to overcome the barriers.

### Problem solving and support

The identification and resolution of barriers and assistance in coming up with implementation plans as well as encouragement were also considered significant tasks. Most of the facilitators agreed that good listening skills were crucial to identify potential problems and to explore the key opportunities for future improvements; however, their individual styles differed. Some facilitators tended to brainstorm with the team where they could network for tools, help, organize, and galvanize their thoughts “….*We spent a significant amount of time with our first on-site visit in [city] doing that - helping the site PI really think about who are the right kinds of people, making suggestions, and helping him follow through with that*” and providing encouragement “…*encouragement, cheerleading, you know, positive kind of modeling*”. Other IE facilitators considered touching base with all team members important to understand the system and review of the implementation plan. Some facilitators also mentioned the need to give recognition for small “wins” and show how this contributed to the big picture “ … *I like to sit and listen to things they're doing, activities they're being involved in, and trying to explain to them how those small tests of change, and all these little things that they are doing help out….*”

Usually the teams were aware of their barriers and most of the facilitators worked with their co-facilitator to gather information from the team to help them overcome the barriers. Some facilitators tended to give suggestions and examples of what other people were doing from other facilities as they were familiar with stroke care across VHA, “*…giving suggestions and examples of what other people were doing really sort of instigated them thinking outside the box*”. Other facilitators focused on working to engage the team during the site visit. One IE coach used the example, “*Trying to meet ER nurse manager on her own turf…trying to see where she was …what her reluctance was,…what she had against the changes that we were implementing and then, trying to get her feedback, …what can we do that would work for you, so we tried to get her involved… because we could change the implementation plan based on her feedback*”.

#### Telephone sessions

Facilitation by telephone touched upon similar tasks that were emphasized during the on-site visits. However, most of the facilitators considered the most important tasks during telephone facilitation to be tracking the status of current process, encouragement of teams’ accomplishment, and goal setting for the next telephone facilitation session (see Table 3). Both types of facilitators reported spending 10%–50% of phone call time directly providing information to teams. During the call, the facilitators helped teams to identify the barriers and explore the solution to resolve them. Some of the facilitators started setting a goal for the next call and encouraged the accomplishments with the intention of creating energy for the whole team to work together toward the final goals. Other facilitators focused on keeping the momentum going in order to achieve aims. Since some facilitators focused on the relationship building while on-site, they were more concerned with moving the teams on their quality improvement progress during the telephone sessions. Some facilitators reported it was difficult to coach using the telephone; “*If they [the team] would have a problem with the spreadsheet, it was hard collaborating from 2000 miles away. Trying to meet, instead of me just going in and fixing something, I would rather teach them how to use it*”.

### Changes in facilitation activities over time

As the facilitators engaged with the clinical teams and each other over time, they noticed that their focus and emphasis changed. The overarching emergent themes revolved around the facilitation experience and the partnership between the Stroke-QUERI and VA-CASE. At the start of the facilitation, there was an emphasis on the barriers and challenges the facilitators experienced with the teams, assessment of the team’s progress, building relationships with, and providing tools to the teams as well as assessing the context in which the teams operated. However, as the project time advanced, the facilitators reported their roles and tasks had shifted. Emergent themes related to tasks over time were encouraging/cheerleading the team, pointing out improvements, and empowering the sites to own the project with the intention they would sustain their efforts. Similar to an individual’s readiness to change behaviors, a clinical team may need more resources and intense effort to plan and implement change at the start and then less as the new behaviors are sustained.

### Challenges of facilitation

The Stroke-QUERI and IE facilitators reported their facilitation challenges. Overall, facilitation challenges were related to the front line clinical teams’ allocated time to work on quality improvement and access to real time quality performance data. The Stroke-QUERI facilitators reported different opinions on the unique challenges with teams. One reported scheduling all team members together for a meeting was challenging. Another thought motivating and engaging the team was difficult. Another noticed tension among the clinical team members at a site and questioned how to best bond the team, establish a good relationship, and communicate. The Stroke-QUERI facilitators noted that the clinical teams’ lack of experience with some technology (e.g., the Microsoft SharePoint site) limited their access to useful resources. As far as feedback from the IE facilitators, one reported the difficulty to keep everyone on the team motivated was due to the clinicians’ busy schedule, personnel loss and turnover. Collaboration from a distance was identified by another IE as a barrier, making it difficult to help the teams solve problems immediately. Finally, one IE reported the lack of immediately available data (quality data was being collected from a central location for the project) was another challenge.

### Emergent themes from facilitators

Overall, the major themes from the facilitators on their roles working with the front line clinical teams were as follows. Most of the facilitators discussed that they saw their role as empowering the clinical teams to take ownership of the process changes at the clinical sites to improve their performance quality. To do so, the facilitators discussed how they spent time with the sites reviewing the teams’ progress and barriers, helping them plan how to overcome the barriers, and following up on progress. Facilitators explained that a good proportion of their time with the clinical teams was spent on listening to the front line staff and providing support and suggestions on how to navigate their plans within their medical facility. The front line staff was usually versed in how to operate within their service or department. However, they often needed suggestions on how to best navigate proposed changes across services and across clinician roles and within their organizational leadership.

### Leadership reflection on collaborative facilitation

Both the leaders of the two programs, Stroke-QUERI and VA-CASE, reported their observations of their planned facilitation implementation strategy. In terms of the perspective of unique contributions, both of VA-CASE and Stroke-QUERI leadership thought that IE facilitators brought the technical perspective to the facilitation process. The Stroke-QUERI leadership felt that it was a structured approach “*I think the addition of the VERC engineers helped us get to some more specifics, helped us provide more targeted tools and strategies to the teams than maybe Stroke-QUERI personnel on their own might not have tended to do…*” Both VA-CASE and Stroke-QUERI leadership thought that Stroke-QUERI facilitators supplied the clinical suggestions and the VA-CASE leadership added that the Stroke-QUERI facilitators “…*bring the people, organizational, cultural perspective*”.

Going forward, the VA-CASE leadership reported the IE facilitators are considered an important facilitation strategy to support changing processes at facilities and one example in which the Stroke-QUERI and VA-CASE partnered successfully. For Stroke-QUERI leadership, combining Stroke-QUERI facilitators and the VA-CASE facilitators together delivered a strong facilitation model and brought new tools and techniques to Stroke-QUERI implementation scientists.

## Discussion

This study provided a qualitative content analysis of the facilitation activities and roles conducted by two groups of external facilitators (Stroke-QUERI and IE facilitators) over the course of a stroke quality improvement project. The facilitators generally identified similar core activities in external facilitation, and worked together to help teams identify and overcome their barriers and assess their progress to achieving their set project goals. Each team brought complimentary experiences and skill sets to the partnership and learned from each other.

Other important experiences identified for future external facilitation efforts include establishing rapport and communication with teams, applying process improvement tools, suggesting solutions and empowering teams to identify sources of help, and keeping teams focused and on track. Our data supports the literature that key roles of external facilitation [27] include fostering support to the field teams through identifying referrals and resources as well as providing guidance [23] through the planned tasks and application of tools [2]. With this relationship established, the usage of tools and changing practices could then be improved. Strong communication practices enabled the facilitators to understand the problems with which the teams struggled and make plans to help within the local context. Our findings support that of Kauth et al. [8] that an important barrier to implementation was team members’ poor communication with clinical leaders, and therefore, communication and team cohesiveness were factors targeted by our external facilitators.

Giving suggestions and examples of solutions to resolve identified problems as well as encouraging teams to learn to solve problems using local or distant expertise was also a core activity. Similar to previous Stroke-QUERI implementation research [2], our external facilitators reported the application of problem solving, use of formative data, motivation to the field, and providing support and encouragement to the front line clinicians involved with practice changes as key aspects of facilitation. External facilitators emphasized that they guided the local teams through the processes rather than just implementing the local practice changes. Finally, keeping the team on task by frequent engagement helped keep the teams focused. Another emerging aspect of external facilitation was that the facilitators noted how their activities changed over time. Early on, engagement and relationship building plus facilitation through the use of tools were paramount. This activity transitioned to more monitoring, problem solving, and intentional work to hand over the clinical improvement process to the site teams with the coach’s role being increasingly that of a more distant consultant. Overall, this study demonstrated that external facilitation is not an event but rather a process where relationships and responsibilities evolve over time.

As found by Rycroft-Malone et al. [4], at times, a lack of a top-down support and lack of accountability for change made the implementation of change a challenge in this project. The clinical teams in this project had volunteered to participate and received no additional resources to implement practice changes. This research also found that sites with external facilitation were appreciative of the time and expertise provided by the facilitators. This finding supports that of Bunniss et al. [17] that the value of the external facilitators became apparent to the participant after the first facilitation session, and their contributions were recognized by teams as valuable.

Our findings also have implications for the application of facilitation in the implementation science conceptual framework, PARiHS [1,3,4]. By using our facilitation elements as inclusion criteria, our findings can determine the appropriateness of the facilitation concept in future applications. Moreover, the facilitation components utilized by our facilitators are aligned with the “holistic” approach to facilitation which enables others to perform and achieve goals in the context of a research, voluntary effort [1]. Future research may determine whether these identified core components of facilitation remain stable across different contexts or whether additional components are deemed necessary for facilitation associated with successful implementation.

## Study limitations

The data reported in these secondary analyses are retrospective; thus, recall may be limited. Additionally, the data is only from the facilitators themselves and not from the teams receiving the aid; therefore, it may not be the entire picture. Since this data is from a relatively small group of external facilitators, generalizability may be limited and differences may be due to individual differences. Because of the small number of facilitators involved, facilitators may have felt unable to speak freely about their experiences out of concern for identification during analysis. To minimize this concern, all interviews were conducted by a qualitative researcher not involved in this intervention and transcripts were de-identified prior to analysis. Nonetheless, this study provided an in-depth description of the external facilitation process from two types of facilitators. Future research should be designed to evaluate the effectiveness of such facilitation.

## Conclusions

From a practical perspective, it is suggested that this external facilitation model may be adopted especially for complex practices and to help move research evidence into practice. This study shows that external facilitation involves core elements related to communication, relationship building, methods training, monitoring performance over time, and facilitating team-based problem solving. Importantly, this work demonstrates the fluid nature of external facilitation over time, as teams learn, grow, change, and experience changing contexts. Stroke-QUERI and IE facilitators use complimentary but somewhat distinct methods to engage clinical teams, and an ideal external facilitation effort would incorporate both aspects. Future studies of external facilitation should consider quantifying these activities and their change over time and explore different modes of conducting the activities to maximize learning and performance while minimizing cost.

## Additional files

Additional file 1:
**Facilitator (coach) survey.** A list of semi-structured questions used to interview the external facilitators at the end of all external facilitation activities to understand their unique and overlapping facilitation skills, knowledge, and contributions as well as identify the critical elements of external facilitation. Additional file 2:
**Leadership survey.** A list of semi-structured questions used to interview the leadership at the end of all external facilitation activities.

## Abbreviations

VA-CASE: Veterans Affairs-Center for Applied Systems Engineering
IE: Industrial engineers
INSPIRE: Intervention for Stroke Improvement using Redesign Engineering
OSE: Operational Systems Engineering
PARiHS: Promoting Action on Research Implementation in Health Services
SR: Systems Redesign
VA: Veterans Affair
VERC: Veterans Engineering Resource Center
VHA: Veterans Health Administration
QUERI: Quality Enhancement Research Initiative
