# Supplementary material for: Key components of external facilitation in an acute stroke quality improvement collaborative in the Veterans Health Administration
Source: Implement Sci. 2015 May 14;10:69. doi: 10.1186/s13012-015-0252-y (PMC4437451; doi:10.1186/s13012-015-0252-y)
Supplement: Additional file 1: — Facilitator (coach) survey. A list of semi-structured questions used to interview the external facilitators at the end of all external facilitation activities to understand their unique and overlapping facilitation skills, knowledge, and contributions as well as identify the critical elements of external facilitation. [file 13012_2015_252_MOESM1_ESM.docx]

**Additional file 1: Facilitator (Coach) Survey**

# INSPIRE Coach

1. Are you a VERC Systems Redesign Coach (SR) or a QUERI Implementation Coordinator? (Circle one)
2. Prior to INSPIRE, how many clinical teams have you previously coached? What was the average length of time you spent coaching a team?
3. Please describe your approach or strategy for coaching teams on Quality Improvement (QI) techniques?
4. What did you consider as the most important tasks you aimed to accomplish during your coaching session ON-SITE Visits? Please describe how you delivered these tasks to any of the INSPIRE site teams to illustrate.
5. During your ON-SITE VISIT coaching sessions, what percentage (0-100%) of time would you estimate you spent doing the following activities:
   1. Monitoring the status of the teams’ progress ______%
   2. Providing information to the teams ______ %
   3. Providing support (being a sound board, providing guidance) _____%
   4. Identifying activities to be done _____%
   5. Providing referrals (connecting people to people, connecting people to information)_____%
   6. Identifying resources ______%

Please provide examples for how you provided each of these activities (a-e) as a coach during INSPIRE.

1. What did you consider as the most important tasks you aimed to to accomplish during your coaching session during PHONE Calls? Please describe how you delivered these tasks to any of the INSPIRE site teams to illustrate.
2. During your telephone coaching sessions, what percentage (0-100%) of time would you estimate you spent doing the following activities:
   1. Monitoring the status of the teams’ progress ______%
   2. Providing information to the teams ______ %
   3. Providing support (being a sound board, providing guidance) _____%
   4. Identifying activities to be done _____%
   5. Providing referrals (connecting people to people, connecting people to information)_____%
   6. Identifying resources ______%
3. What percentage of time from 0-100% would you say you used the System Redesign tools with your INSPIRE site teams during the 6 months of coaching?
   1. Project Charter
   2. Process Map
   3. Spaghetti Diagram
   4. Impact Effort Matrix
   5. PDSA
4. Compare this experience on INSPIRE to your other coaching experience(s), explain what are the similarities and differences?
5. How did you assess the teams’ needs (e.g., what did the team need to do next?)
6. How did you respond to the teams’ assessed needs (e.g., how did you assist the teams in facilitating changes at their site?)
7. How did you help teams identify their barriers to change? (please provide examples to illustrate)
8. How did you help teams overcome their barriers to change? (please provide examples to illustrate)
9. During your coaching sessions:
   1. What were some of the unique challenges you experienced in coaching teams?
   2. What were the unique challenges you experience in coaching with other (VERC/QUERI) coach?
   3. What are the greatest satisfactions you experience in coaching with other (VERC/QUERI) coach?
   4. What did you learn from the VERC/QUERI Coach?  Please give examples.
   5. How did you handle any disagreement with the VERC/QUERI Coach?
10. What steps, if any, do you take to ensure teaching QI techniques to teams was spread with other team members who did not attend the SR training?
11. If you could make one change that would help improve teaching QI techniques to teams what would it be?
12. What are some of the barriers or constraints that you experienced as a coach? Please list some examples.
13. Anything you’d like to add?

**APPENDIX 2: Leadership Survey**

## INSPIRE/VERC Leadership – Please circle one

1. **What was your overall expectation for VERC/QUERI coaching together prior to the start of INSPIRE?**
2. **What expectations were met? How so?**
3. **What expectations were not met? How so?**

**4. What do you think was unique about the contribution from the QUERI coaches?**

**5. What do you think was unique about the contribution from the VERC coaches?**

**6. Did you learn anything new during your experience coaching on this project? If so, what was it?**

**7. How would you design a future VERC/QUERI collaboration for Quality Improvement (QI) based upon your knowledge of INSPIRE?**
