# Supplementary material for: Key components of external facilitation in an acute stroke quality improvement collaborative in the Veterans Health Administration
Source: Implement Sci. 2015 May 14;10:69. doi: 10.1186/s13012-015-0252-y (PMC4437451; doi:10.1186/s13012-015-0252-y)
Supplement: Additional file 2: — Leadership survey. A list of semi-structured questions used to interview the leadership at the end of all external facilitation activities. [file 13012_2015_252_MOESM2_ESM.doc]

**Additional file 2: Leadership Survey**

## INSPIRE/VERC Leadership – Please circle one

1. What was your overall expectation for VERC/QUERI facilitation together prior to the start of INSPIRE?
2. What expectations were met? How so?
3. What expectations were not met? How so?

4. What do you think was unique about the contribution from the QUERI facilitators ?

5. What do you think was unique about the contribution from the VERC facilitators ?

6. Did you learn anything new during your experience facilitation on this project? If so, what was it?

7. How would you design a future VERC/QUERI collaboration for Quality Improvement (QI) based upon your knowledge of INSPIRE?
